# Roles of Non-Coding RNA in Sugarcane-Microbe Interaction

**DOI:** 10.3390/ncrna3040025

**Published:** 2017-12-20

**Authors:** Flávia Thiebaut, Cristian A. Rojas, Clícia Grativol, Edmundo P. da R. Calixto, Mariana R. Motta, Helkin G. F. Ballesteros, Barbara Peixoto, Berenice N. S. de Lima, Lucas M. Vieira, Maria Emilia Walter, Elvismary M. de Armas, Júlio O. P. Entenza, Sergio Lifschitz, Laurent Farinelli, Adriana S. Hemerly, Paulo C. G. Ferreira

**Affiliations:** 1 Laboratório de Biologia Molecular de Plantas, Instituto de Bioquímica Médica Leopoldo de Meis, Universidade Federal do Rio de Janeiro, Rio de Janeiro 21941-901, Brazil; flaviabqi@gmail.com (F.T.); treecko_blaziken@hotmail.com (E.P.d.R.C.); mariana.attom@gmail.com (M.R.M.); helfos85@gmail.com (H.G.F.B.); babicp@hotmail.com (B.P.); berenicenagelasl@gmail.com (B.N.S.d.L.); hemerly@bioqmed.ufrj.br (A.S.H.); 2 Universidade Federal da INTEGRAÇÃO Latino-Americana, Foz do Iguaçu 85866-000, Brazil; cristian.rojas@unila.edu.br; 3 Laboratório de Química e Função de Proteínas e Peptídeos, Universidade Estadual do Norte Fluminense, Campos dos Goytacazes 28013-602, Brazil; cgrativol@uenf.br; 4 Departamento de Ciência da Computação, Universidade de Brasília, Brasília 70910-900, Brasil; maciel.lucas@outlook.com (L.M.V.); mariaemilia@unb.br (M.E.W.); 5 Departamento de Informática, Pontifícia Universidade Católica do Rio de Janeiro, Rio de Janeiro 22451-900, Brazil; earmas@inf.puc-rio.br (E.M.d.A.); jentenza@inf.puc-rio.br (J.O.P.E.); sergio@inf.puc-rio.br (S.L.); 6 Fasteris SA, 1228 Plan-les-Ouates, Switzerland; ht_seq@fasteris.com

**Keywords:** microRNA, *Acidovorax avenae*, pathogen, diazotrophic bacteria, siRNA

## Abstract

Studies have highlighted the importance of non-coding RNA regulation in plant-microbe interaction. However, the roles of sugarcane microRNAs (miRNAs) in the regulation of disease responses have not been investigated. Firstly, we screened the sRNA transcriptome of sugarcane infected with *Acidovorax avenae*. Conserved and novel miRNAs were identified. Additionally, small interfering RNAs (siRNAs) were aligned to differentially expressed sequences from the sugarcane transcriptome. Interestingly, many siRNAs aligned to a transcript encoding a copper-transporter gene whose expression was induced in the presence of *A. avenae*, while the siRNAs were repressed in the presence of *A. avenae*. Moreover, a long intergenic non-coding RNA was identified as a potential target or decoy of miR408. To extend the bioinformatics analysis, we carried out independent inoculations and the expression patterns of six miRNAs were validated by quantitative reverse transcription-PCR (qRT-PCR). Among these miRNAs, miR408—a copper-microRNA—was downregulated. The cleavage of a putative miR408 target, a laccase, was confirmed by a modified 5′RACE (rapid amplification of cDNA ends) assay. MiR408 was also downregulated in samples infected with other pathogens, but it was upregulated in the presence of a beneficial diazotrophic bacteria. Our results suggest that regulation by miR408 is important in sugarcane sensing whether microorganisms are either pathogenic or beneficial, triggering specific miRNA-mediated regulatory mechanisms accordingly.

## 1. Introduction

Sugarcane is an economically important crop for sugar and ethanol production [1]. Current commercial varieties of sugarcane are hybrids derived from crossings among *Saccharum officinarum*, *S. sinense*, *S. barberi*, *S. robustum*, and *S. spontaneum* species [2]. Sugarcane also has one of the most complex plant genomes, having at least 10 copies of most homologous loci [3], consequently hindering the complete sequencing of the sugarcane genome. In addition, crop productivity is negatively affected by environmental stress conditions, such as biotic stress induced by pathogenic bacteria [4]. Among important pathogens of sugarcane, *Acidovorax avenae* subsp. *avenae* affects crops practically worldwide [5]. This bacterium was previously classified as Pseudomonas, and recently reclassified to the Acidovorax genus [6]. In sugarcane, *A. avenae* causes the Red Stripe Disease, which damages leaves and leaf sheaths [7]. Despite of the harm caused by the disease, little is known about the molecular mechanisms triggered in sugarcane in response to the infection. 

Studies established the role of plant small RNAs (sRNAs) in biotic and abiotic stress responses [8,9]. In plants, sRNAs are non-coding RNAs involved in gene expression regulation and can be divided into two categories: microRNA (miRNA) and small interfering RNA (siRNA), both produced by RNase III-like enzymes called DCLs—Dicers-like [10,11]. The siRNA can be divided into three subclasses, heterochromatic siRNA (hc-siRNA—derived from repeat sequence), natural antisense siRNA (nat-siRNA), and trans-acting siRNA (ta-siRNA—its biogenesis is dependent on miRNAs). However, differences between siRNA and miRNA include the kind of precursor, enzymes involved in biogenesis, and gene silencing mechanisms [12]. Mature miRNAs regulate protein-coding genes post-transcriptionally by mediating RNA cleavage or translational repression [13]. However, the most frequent mechanism of plant miRNA regulation is direct cleavage of the mRNA-target [14]. Several targets of miRNAs are genes that play important roles in plant responses to biotic stress. For instance, miR393 targets auxin receptor genes, such as TIR1 (Transport Inhibitor Response 1), AFB2 (Auxin Signaling F-Box2), and AFB3, attenuating auxin signaling and inhibiting infection by *Pseudomonas syringae* [15]. MicroRNAs are also induced by pathogenic or symbiotic bacteria, suggesting the involvement of miRNAs in plant-microorganism interactions such as defense or symbiosis [16,17,18,19].

Long non-coding RNAs (lncRNAs) are a class of non-coding RNAs that have emerged as important regulators of gene expression. lncRNA are transcripts of more than 200 nucleotides in length, and those occurring with intergenic spaces are classified as long intergenic non-coding RNAs (lincRNAs) [20,21,22]. Despite the knowledge of the role of miRNAs in plants, little is known about the function of lncRNAs. However, some studies have identified lncRNAs as differentially regulated in response to biotic and abiotic stress [23,24,25].

In order to elucidate how sRNAs are implicated in the responses to pathogenic bacteria in sugarcane, we first employed next-generation sequencing technology to uncover the global regulation of the sRNA transcriptome of sugarcane plants infected with *A. avenae* subsp. *avenae*. Using this approach, we identified novel sugarcane miRNAs and conserved miRNA families. Moreover, we aligned siRNAs in the sugarcane transcriptome to observe the putative regulation of genes by siRNA. Interestingly, a transcript annotated as a copper-transporter exhibited many aligned siRNAs. The expression profiles of the siRNAs and the transcript are inversed. In addition, 67 sugarcane lincRNAs were identified, and one of these aligned with miR408. Bioinformatics analysis of the libraries indicated that copper-microRNAs—i.e. microRNAs that regulate mRNAs encoding copper-binding enzymes [26,27,28]—are differentially regulated by *A. avenae*. In order to confirm this observation, we selected two copper-microRNAs to verify their expression profiles in four replicates from an independent experiment. In all samples, miR408 and miR397 were repressed in plants infected with pathogenic bacteria. Furthermore, the downregulation of miR408 expression was also observed in plants infected with the pathogenic fungus *Puccinia kuehnii*, the causal agent of orange rust, but not in plants inoculated with beneficial bacteria. MiR408 regulates a laccase and the inverse expression profile of the miRNA and its target was confirmed, as well the cleavage mediated by miR408. Our findings suggest an important role for miR408 during sugarcane-microbe interaction. Overall, our results indicate that the plant’s ability to distinguish beneficial from pathogenic bacteria is determined, at least in part, by the ability to induce the expression of specific sRNAs.

## 2. Results

### 2.1. Pathogen Assay and Small RNA Sequencing

**Figure 1:**
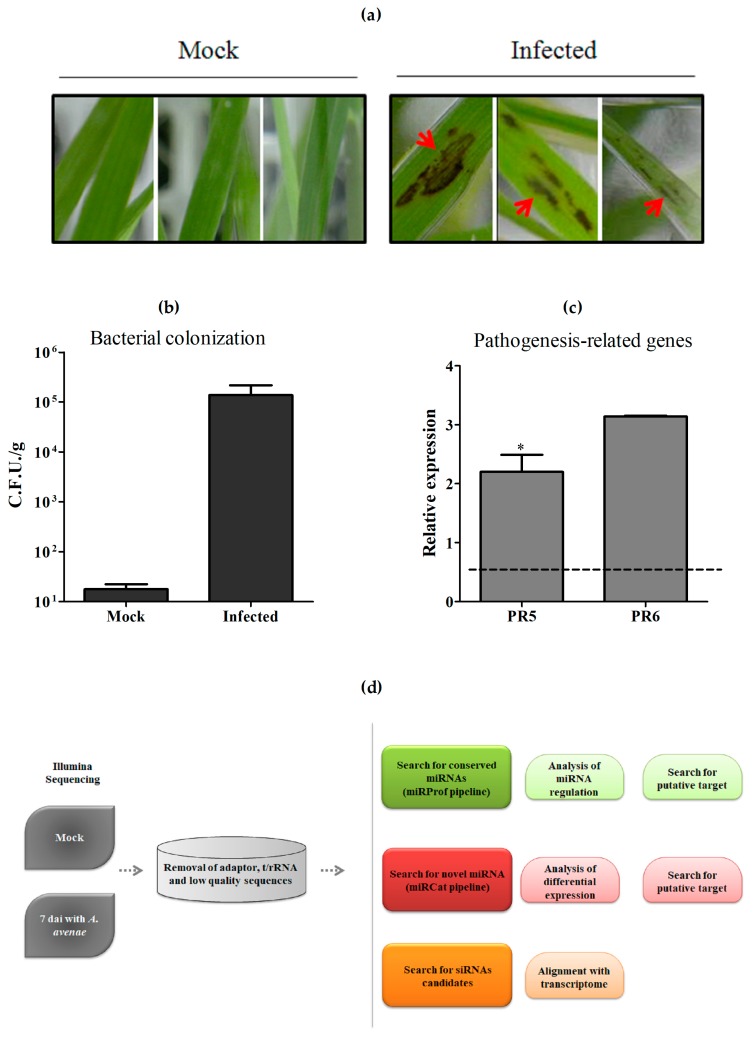
Plant treatment and analysis pipeline employed. (**a**) Photograph of sugarcane plants 7 days after inoculation with *Acidovorax avenae.* Red arrows show spots present in all infected plants, but not in control mock-inoculated plants; (**b**) Counts of bacterial colonization by MPN (Most Probable Number) estimation using mock and infected plants after 7 days of inoculation with *A. avenae*. The values show the average bacterial number counted in two experiments; (**c**) Analysis of two pathogenesis-related genes, PR5 and PR6, in response to the inoculation of *A. avenae* using real-time PCR. The error bars represent the standard deviation between three technical replicates. * represents significant changes of target gene expression between control and inoculated samples (*p*-value < 0.05). Dashed line represents the control; (**d**) Pipeline followed to analyze sugarcane sRNA from mock-inoculated and pathogen-infected samples. C.F.U.: Colony Forming Units.

In order to obtain a panorama of sRNA regulation in response to pathogenic infection in sugarcane, we firstly constructed sugarcane sRNA libraries using RNAs from sugarcane plantlets grown in vitro and inoculated with the bacterium *A. avenae* subsp. *avenae*. Seven days after the inoculation (dai), plants were harvested for RNA extraction and two biological replicates (Rep 1 and Rep 2) were used for library construction and sequencing. Infected plants showed conspicuous dark spots in all experiments, as shown in Figure 1a. Moreover, colonization was also confirmed by bacterial counting using plants 7 dai (Figure 1b). Because a pathogen attack triggers a complex molecular response in plants and pathogenesis-related (PR) proteins are crucial components of the plant’s self-defense mechanisms [29], we verified the expression of two PR genes, PR5 and PR6, in infected plants from Rep 1. Both genes were induced, confirming that defense responses were triggered by treatment with *A. avenae* (Figure 1c). In addition, analysis of mRNAseq obtained from RNA extracted from the same samples uncovered plenty of defense pathways [30].

Following the protocols established in these initial experiments, sugarcane sRNA libraries were constructed using RNAs from plants either infected with the *A. avenae* or mock-inoculated. Small RNAs ranging from 20 to 28 nucleotides (nt) in length were selected for further analyses. The bioinformatics pipeline used is shown in Figure 1d. First, adapters, contaminants, t/rRNA, and low-quality reads were removed, yielding 1,560,245 and 1,436,225 reads from mock and infected libraries, respectively (Table 1). The reads obtained from libraries of Rep 2 are given in Table S1. Conserved miRNAs were identified based on matches with the miRBase database, release 21, allowing three mismatches. Using the miRProf pipeline [31], 1872 and 2313 unique sequences of conserved miRNAs were identified in mock and infected libraries of Rep 1 (Table 1). The remaining reads can be classified as putative siRNA or novel miRNA.

### 2.2. Sugarcane lincRNAs

In order to identify the putative sugarcane lincRNAs, a specific pipeline was developed (Figure S1). Out of the total of 168,767 sugarcane transcripts, 65,419 transcripts were not identified as protein-coding (using Basic Local Alignment Search Tool (BLAST) with sugarcane transcripts and a database containing protein-coding Sorghum transcripts). However, from these 65,419 transcripts, 2030 were identified as protein-coding by BLAST when using protein-coding transcripts of the Panicoidae subfamily from the database Repbase. Thus, after removing protein-coding transcripts, 63,389 transcripts were further analyzed, of which 1466 were identified as lncRNAs using BLAST, with these transcripts as queries against the lncRNAs transcripts in the CantataDB database. 

In the mapping step, 9488 protein-coding transcripts from sugarcane were mapped onto sorghum gene regions, with 2687 non-coding transcripts mapped in intergenic regions. For the sugarcane support vector machines (SVM) model, 12 features were used: open reading frame (ORF) length; ORFs’ proportion; and 10 di- and tri-nucleotides frequencies (AA, AT, CA, CC, CG, GA, GC, GG, TG, TT) identified by Principal Component Analyses (PCA). To train the model, positive and negative datasets of 1000 transcripts each were built. From 2432 sugarcane transcripts mapped on intergenic regions, 1689 were classified as lincRNAs by the SVM model and 97 by BLAST [32]. Finally, a total of 67 transcripts were classified as lincRNAs by both BLAST and the SVM model. Table S2 shows the results obtained from this analysis.

**Figure 2:**
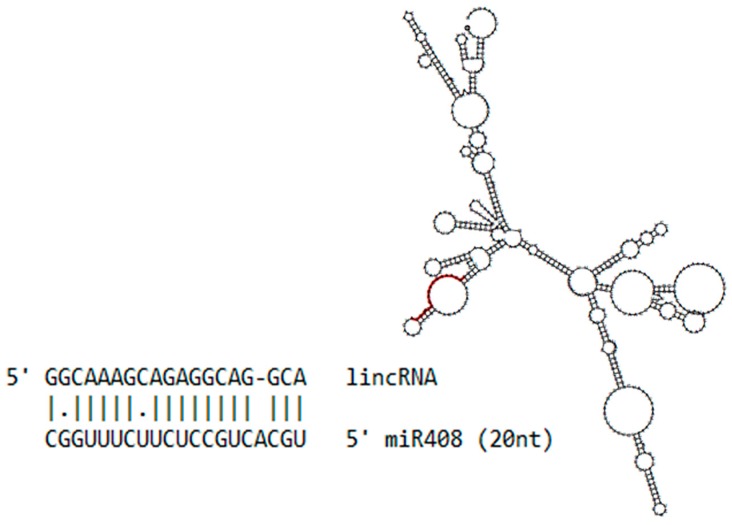
Long intergenic non-coding RNAs (lincRNA) and miR408. The secondary structure of identified sugarcane lincRNA, with the position of the aligned miR408 highlighted in red, as well as the alignment of these two sequences showing the gap and mismatches.

One lincRNA has noteworthy complementarity with miR408 (Figure 2). This lincRNA is 613 nt in length and aligns with 20 nt of the miR408 sequence, suggesting that this lincRNA could potentially act as a miRNA decoy or miRNA target, inhibiting the regulation of the canonical miR408 target. 

### 2.3. Novel Sugarcane miRNAs Were Identified

**Figure 3:**
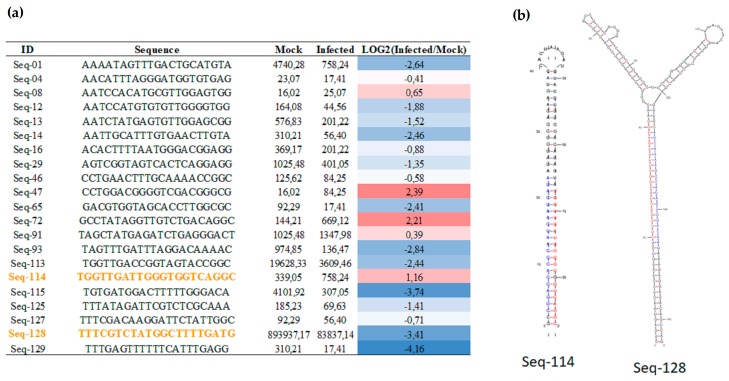
Novel miRNA present in both libraries. (**a**) Differential expression levels of novel miRNAs with more than 25 raw reads present in both libraries. The raw numbers were normalized to reads per million and compared between libraries from mock and infected plants. The log2 transformation counts were performed in the infected/mock comparisons. The heatmap showed miRNAs to be downregulated (blue) and upregulated (red) in response to pathogenic infection. Novel miRNA that have miRNA* in the same library were highlighted by orange letters; (**b**) Bona fide precursors; mature miRNAs (red) and miRNA* (blue).

A previous study identified 384 novel sugarcane miRNA regulated in response to pathogenic infection [33]. However, these earlier analyses were conducted using the Sorghum genome database. A new search against the sugarcane genome sequencing by methylation filtration database [34] identified additional 131 new sugarcane miRNAs, 21 and 22 nt in length, from mock and infected libraries using the miRCat pipeline (Table S3). The MFEI (Minimum Fold Free Energy Index), another parameter used to evaluate the novel miRNA precursors, was calculated manually according to Zhang et al. (2006) [35]. More than 82% of new miRNAs had a MFEI value higher than 0.7 (Table S3), robust evidence that these sequences are miRNA precursors. Twenty-one novel miRNAs showing differential expression were identified in both libraries (Figure 3a). The expression levels were normalized to compare the expression in all libraries. Interestingly, two novel miRNAs had both mature miRNA and miRNA* identified in the same library (Table S3; Figure 3a,b). Using a psRNA target, it was possible to identify putative targets for 84 novel miRNAs in sugarcane (Table S4). In this analysis, the Expressed Sequence Tags (EST) sugarcane data from Gene Index version 3.0 was used and it was possible to observe that some targets are involved in defense response. For instance, the Seq-114 is upregulated and, the putative target is a peroxidase (Table S4). 

### 2.4. Sugarcane siRNA Were Identified

**Figure 4:**
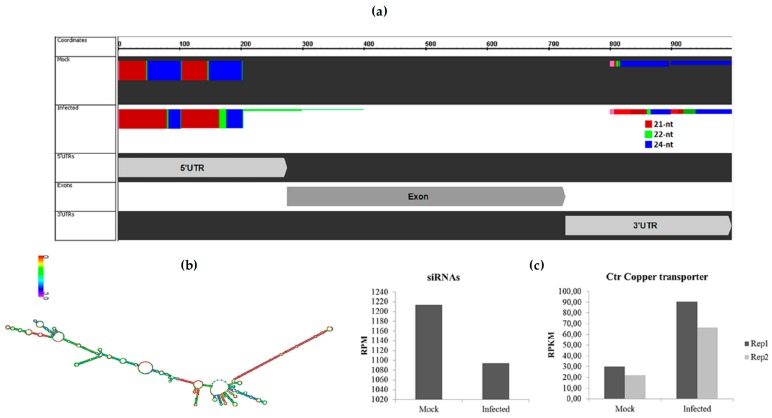
siRNAs regulate copper transporter. (**a**) Alignment of a cluster of siRNAs from sRNA libraries in Ctr copper transporter. Colors represent sRNAs with different lengths; (**b**) Structure of the Ctr copper transporter transcript. The long hairpin in red is the 5′ UTR region; (**c**) Expression levels of siRNA cluster that aligned with Ctr copper transporter from sRNA libraries, and the expression of Ctr copper transporter using data from the transcriptome of sugarcane infected with *A. avenae* and mock-inoculated. Two biological replicates of these transcriptomes were used [30]. RPM: Reads Per Million; RPKM: Reads Per Kilobase Million.

After removing novel and conserved miRNAs sequences, the siRNAs candidates were identified. In order to classify these siRNAs, candidate sequences were aligned with differentially expressed transcripts from a transcriptome of sugarcane infected with *A. avenae*. A total of 110 differentially expressed transcripts showed more than 30 aligned siRNAs in at least one library (Table S5). Interestingly, one of them is a transcript annotated as copper transporter (Ctr). The alignment of siRNAs and this transcript is depicted in Figure 4a. Curiously, in the mock samples 42% of siRNAs that aligned with the copper transporter have 24 nt, followed by 37% with 21 nt in length; while the distribution of the siRNA length in the infected library was 41% for each size, 21 nt and 24 nt. In addition, the result showed that the majority of siRNA aligned in the 5′ UTR—untranslated region, with siRNAs also aligning in the 3′ UTR. Curiously, in the predicted RNA structure of this transcript (prediction was made using RNAfold WebServer [36], the 5′ UTR forms a stem-loop structure reminiscent of a miRNA precursor (Figure 4b). In addition, the expression profile of siRNAs that aligned with this transcript was determined, and their downregulation was observed in sugarcane infected with *A. avenae* (Figure 4c). On the other hand, analysis in the transcriptome database revealed that the Ctr copper transporter was upregulated in response to *A. avenae* infection (Figure 4c). 

### 2.5. Differential Expression of Conserved Sugarcane miRNAs in Response to A. avenae Infection

In order to identify the sugarcane miRNAs induced by *A. avenae* infection, the miRNA sequences were grouped into conserved miRNA families. The expression profile of each miRNA was then calculated using the miRProf pipeline and the expression levels were normalized to compare the expression in all libraries. Similarity searches, using a filter of a minimum of 35 reads in at least one library, identified 25 families of conserved miRNAs (Table S6). Among these, the most abundant miRNA family was miR397, followed by miR159 and miR408. A total of 12 miRNA*s had detectable expression (Table S6). 

Interestingly, the expression profiles of mock and pathogen-infected libraries showed dynamic miRNA regulation in response to infection (Table S6). Similar expression profiles in Rep 1 and Rep 2 analysis were observed for 10 miRNAs (Figure S2). For instance, miR156, miR164, miR444, miR528, and miR827 were upregulated, while miR166, miR166*, miR167*, miR408, and miR408* were downregulated. Although the variation on miRNAs expression between replicates is present, there is a very good correlation (cor > 0.82) (Figure S3).

**Figure 5:**
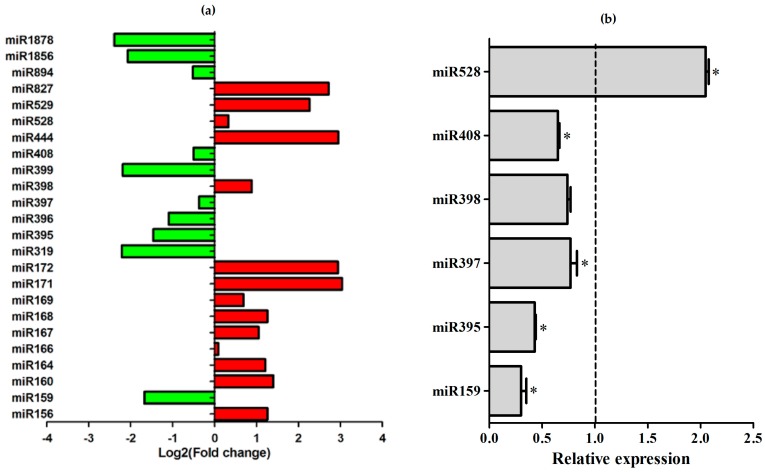
Expression analysis of the miRNAs in response to bacterial infection. (**a**) miRNA expression levels from bioinformatics analysis; (**b**) Validation of bioinformatics expression profiles of selected miRNAs by real-time PCR, using Rep 1 samples. The expression of six miRNAs was tested in mock-inoculated plants and *Acidovorax avenae*-infected plants. Dotted line represents the expression of the control samples. The error bars represent the standard deviation between three technical replicates. * represents significant changes of target expression between the control and inoculated samples (*p*-value < 0.05).

Remarkably, the analysis from Rep 1 showed that three out of the four copper-miRNA families—miR397, miR398, and miR408—were downregulated in response to pathogen infection. Figure 5a shows the expression profile of miRNA families in response to *A. avenae* infection. 

### 2.6. microRNAs Exhibiting Similar Regulation in Response to Different Pathogens

To confirm the expression patterns of miRNAs identified by bioinformatics in the libraries, six miRNAs were selected to be analyzed by stem-loop Reverse Transcription Polimerase Chain Reaction (RT-PCR). Three miRNAs—miR408, miR397, and miR398—which were previously characterized as involved in copper homeostasis (copper-miRNA), were selected for this validation. In addition, three other miRNAs—miR159, miR395, and miR528—were selected randomly to confirm their regulation in plants infected with *A. avenae*.

The expression profiles of most of these miRNAs were confirmed by stem-loop RT-PCR analysis (Figure 5b). One exception was miR398, which in the bioinformatics analysis was upregulated, while in Rep 1 miR398 levels showed a tendency to be repressed in the quantitative Reverse Transcription-PCR (qRT-PCR) analysis. In contrast, analysis of Rep 2 samples showed the downregulation of miR398, confirming the bioinformatics analysis (Figure S2). MiR398, which is regulated in response to biotic stress [37], is a copper-miRNA like miR397 and miR408. The analysis of miR397 expression confirms the bioinformatics analysis from Rep 1, which showed that this miRNA was downregulated under pathogen infection (Figure 5). Once more, miR408 levels decreased in the infected samples, similar to what was observed in the libraries (Figure 5 and Figure S2). Similar expression profiles were observed for miR159 and miR395, suggesting that all of these miRNAs were downregulated in response to pathogenic infection. On the other hand, miR528, also a copper-miRNA, was upregulated in the bioinformatics analysis of plants infected with *A. avenae*, and this expression profile was confirmed by qRT-PCR analysis (Figure 5). 

**Figure 6:**
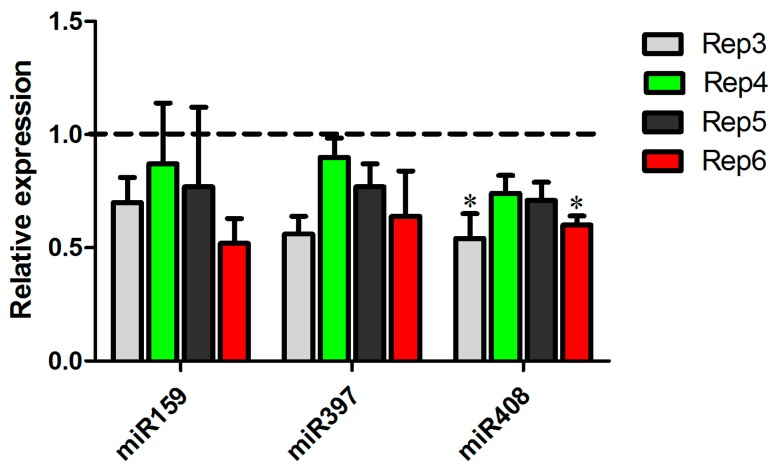
Validation of miRNA expression in the other four experiments. The expression of miR159, miR397, and miR408 was tested in mock-inoculated plants and *Acidovorax avenae*-infected plants by real-time PCR. Dotted line represents the expression of the control samples. The error bars represent the standard deviation between three technical replicates. * represents significant changes of target expression between the control and inoculated samples (*p*-value < 0.05).

The expression patterns observed in the biological replicas sequenced showed an intriguing regulation of the copper-miRNAs. To confirm these observations, we performed an independent experiment of inoculation with *A. avenae* and prepared RNA from four replicates. The expression profile of two copper-miRNAs, miR397 and miR408, as well as miR159 were validated (Figure 6). The results showed that there is a tendency of reduction of miR397 and miR159 expression and a clear repression of miR408 in sugarcane infected with *A. avenae*, indicating the important role of this miRNA in plant defense.

**Figure 7:**
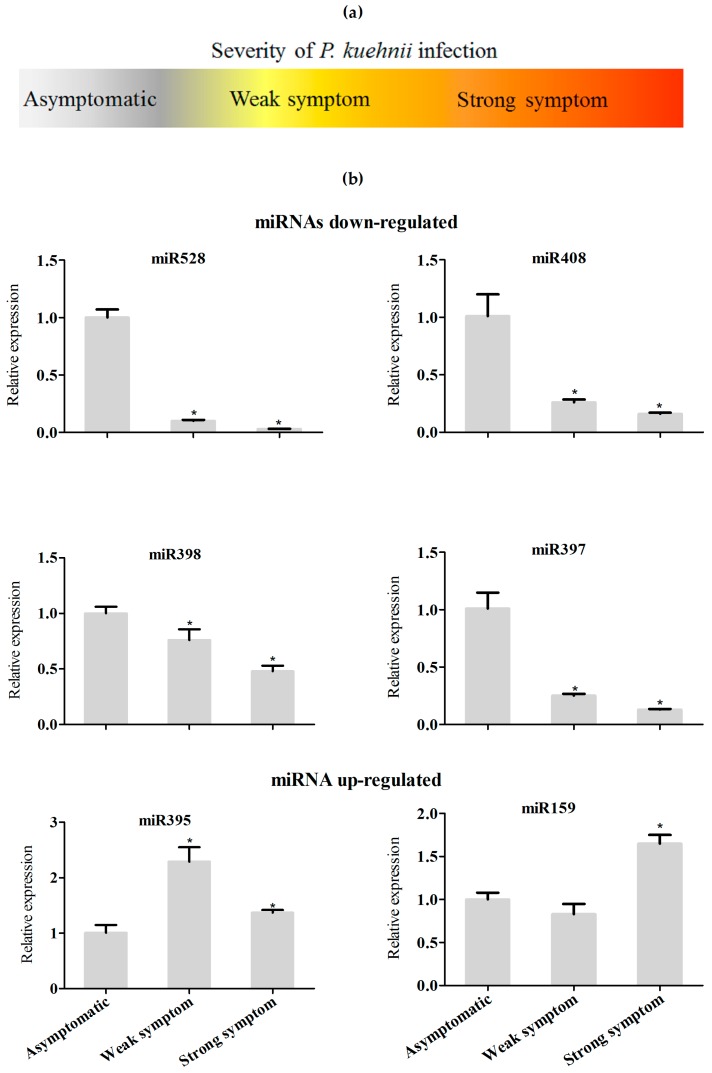
Expression analysis of selected miRNA in sugarcane plants infected with *Puccinia kuehnii*. (**a**) Severity of the disease symptoms in *P. kuehnii* infected sugarcane plants used analyzed; (**b**) miRNAs levels in asymptomatic plants and in *P. kuehnii*-infected plants, assayed by real-time PCR. Statistics were calculated between control and inoculated treatments. The error bars represent the standard deviation between three technical replicates. * represents significant differences in expression between the control and infected samples (*p*-value < 0.05).

Next, we investigated the regulation of miRNAs in response to another important sugarcane pathogen, the fungus *Puccinia kuehnii*, which is the causal agent of orange rust [38]. Because orange rust can cause serious damage to sugarcane crops, there are severe sanitarian restrictions of plant movement from regions with identified infections. Therefore, plants that showed low or high symptoms of *P. kuehnii* infection, in addition to asymptomatic plants, were used for RNA extraction, and the relative expression of miR159, miR395, miR397, miR398, miR408, and miR528 was evaluated (Figure 7a,b). Most miRNA expression did not change in samples from plants exhibiting either weak or strong symptoms of *P. kuehnii* infection, compared to those of from asymptomatic plants. Remarkably, miR397 and miR408 were both downregulated in plants infected with the fungus, similar to what was observed with plants infected with *A. avenae*. Furthermore, miR398 was downregulated in samples infected with *P. kuehnii*, similar to the bioinformatics analysis of Rep 2 and qRT-PCR using *A. avenae* (Figure S2). These results suggest that a pathway mediated by the regulation of copper-miRNA expression might be a shared response to infection with different pathogens.

However, for other miRNAs, we could observe contrasting expression patterns in plants infected with either *A. avenae* or *P. kuehnii* (Figure 5 and Figure 7). For instance, miR159 was strongly downregulated in plants inoculated with *A. avenae*, but was only slightly downregulated in plants infected with *P. kuehnii* that showed weak symptoms. Moreover, miR159 expression was induced in plants with strong symptoms of orange rust disease. These differences between the miRNAs’ regulation could be due to specific responses to bacteria and fungi pathogen, or to the fact that the *A. avenae* experiment was performed with sugarcane plantlets grown in vitro, while samples infected with *P. kuehnii* were harvested from field-grown plants. Sugarcane has a very complex genetic system with a highly polyploid genome. In addition, it is also vegetatively propagated and therefore naturally colonized by many different bacteria and fungi [39]. In order to obtain plants free of microbes one has to resort to in vitro propagated plants, which are heterogeneous, increasing the difficulty of matching growing conditions. Still, it is remarkable that in samples of different origins—in vitro and field-grown plants—infected with different pathogens—bacteria and fungi—copper-miRNAs respond in similar ways.

### 2.7. Differential Regulation of miR408 in Sugarcane Infected with Pathogens or Beneficial Bacteria

MiR408 seems to have an important role in the regulation of plant-microbe interactions, since it was downregulated in plants infected with different pathogens and has the potential to be regulated by a lincRNA decoy. Based on these interesting features, miR408 was chosen for further investigation. 

**Figure 8:**
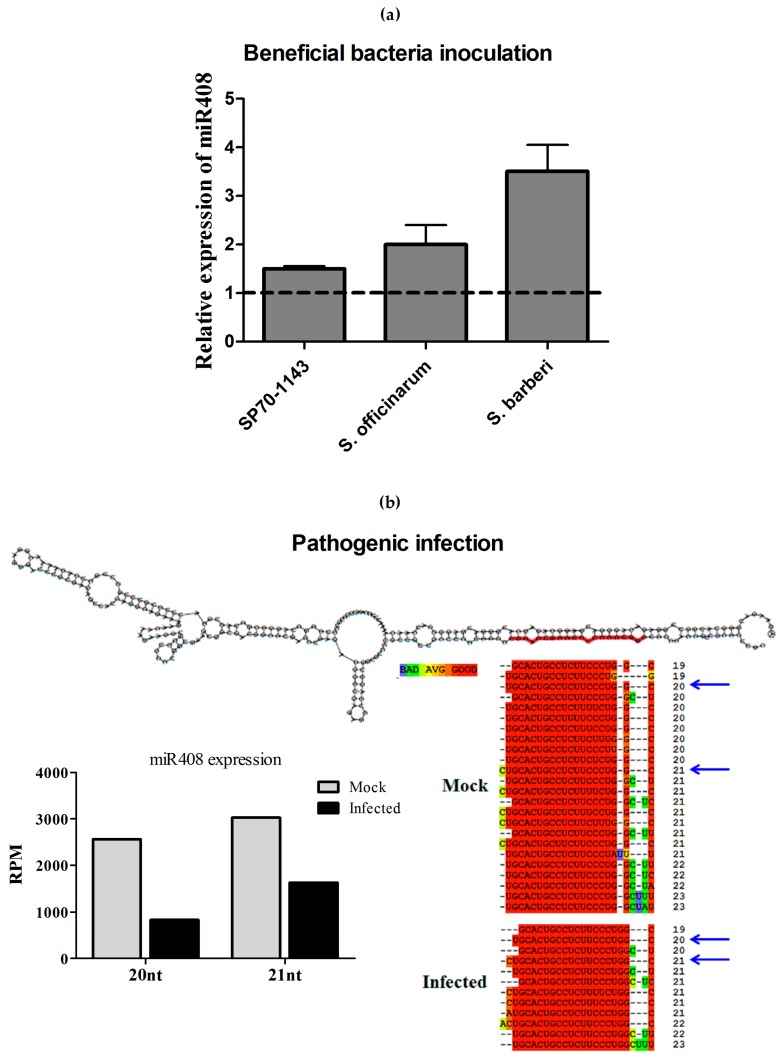
Analysis of miR408-regulation. (**a**) Sugarcane hybrid SP70-1143 and two wild species were used to verify the relative expression of miR408 in response to inoculation with the beneficial diazotrophic bacteria *Gluconacetobacter diazotrophicus* (GD) using quantitative Reverse Transcription-PCR (qRT-PCR). The error bars represent the standard deviation between three technical replicates. * represents significant differences in expression between the control and inoculated samples for each experiment (*p*-value < 0.05); (**b**) Different members of the miR408 family aligned in the same position at their precursor and in the expression profiles of the two more expressed sequences obtained from bioinformatics analysis. These mature sequences were found in mock and infected libraries, and the blue arrow shows the more abundant sequences.

To investigate whether the change in miR408 expression was a unique response to pathogen infection, the expression of miR408 was also quantified in sugarcane plants inoculated with the beneficial endophytic diazotrophic bacteria *Gluconacetobacter diazotrophicus.* SP70-1143 plants, the same hybrid used in the pathogenic assays, were inoculated with *G. diazotrophicus*. At 14 dai, the levels of miR408 were measured by stem-loop qRT-PCR. The results showed that, while miR408 is downregulated in response to pathogenic infection, it was upregulated upon inoculation with *G. diazotrophicus* (Figure 8a,b). This pattern of expression was also seen with two sugarcane wild species, *S. barberi* and *S. officinarum*, inoculated with *G. diazotrophicus* (Figure 8a). Additionally, through semi-quantitative RT-PCR, we also confirmed the downregulation of miR408 in these sugarcane lines upon infection with different pathogens (Figure S4). 

Due the consistent downregulation of miR408 in the presence of sugarcane pathogens, but not in the presence of beneficial bacterial, further analyses were performed to identify and investigate the expression of the putative miR408 targets. First, we confirmed that the mock-infected library had more members of the miR408 family than the infected library, all of them mapping in the same position of the MIR408a precursor deposited in the miRBase database (Figure 8b). The two highest expressed mature miR408 forms are 20 and 21 nt in length. In the bioinformatics analysis, both species were repressed in plants infected with *A. avenae* (Figure 8b). 

**Figure 9:**
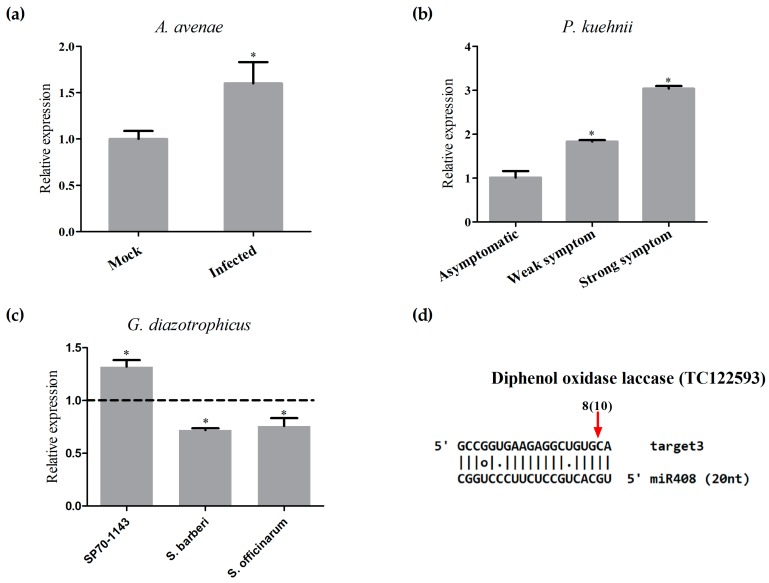
Regulation of laccase by miR408. (**a**) mRNA levels of the putative target of miR408 was analyzed by real-time PCR in mock-inoculated plants, *Acidovorax avenae*-infected plants; (**b**) using *Puccinia kuehnii*-infected plants that showed different degrees of symptoms in comparison to the expression in asymptomatic plants; and (**c**) using sugarcane hybrid SP70-1143 and two wild species inoculated with a beneficial diazotrophic bacteria, *Gluconacetobacter diazotrophicus*. Dotted line represents the expression of the control samples. The error bars represent the standard deviation between three technical replicates. * represents significant differences in expression between the control and inoculated samples for each experiment (*p*-value < 0.05); (**d**) Alignment of miR408 and its putative target. The red arrow shows the cleavage site confirmed by 5′ RACE (Rapid Amplification of cDNA Ends), and the number is the amount of clones that present this cleavage site (in parentheses) in a total of clones that were sequenced.

Data from the literature indicate that miR408 directs the cleavage and downregulation of mRNAs encoding plastocyanin-like proteins [40] and laccases [26]. In order to analyze the biological importance of the sugarcane miR408, we searched for the two putative targets of this miRNA using the psRNAtarget prediction pipeline. The targets are predicted to encode a diphenol oxidase laccase (TC122593), and blue copper protein (plastocyanin-like protein) (TC121661), and are orthologs of two Arabidopsis miR408 targets (At5g05390 and At2g02850), which were validated by 5′RACE (Rapid Amplification of cDNA Ends) PCR [26]. To investigate whether the expression of mature miR408 and its target were inversely correlated in sugarcane plants treated with pathogens, the two putative targets were selected for expression analysis by qRT-PCR, using samples of RNA extracted from sugarcane infected with *A. avenae* and *P. kuehnii*. In samples of sugarcane infected with either *A. avenae* or *P. kuehnii*, the expression of TC122593 was inversely correlated with the measured levels of miR408 (Figure 9a,b), suggesting that the laccase mRNA is a target for miR408 in sugarcane. The other putative target, TC12661, also was upregulated in sugarcane infected with both pathogens (Figure S4). Based on the differential regulation of miR408 in response to different microbes, the expression of miR408 targets was also verified in samples of sugarcane infected with a beneficial diazotrophic bacterium. TC12661 was repressed in all sugarcane plants inoculated with *G. diazotrophicus* (Figure S5). However, a repression of TC122593 was observed only in two wild species of sugarcane, *S. barberi* and *S. officinarum*, inoculated with *G. diazotrophicus* (Figure 9c). Unfortunately, given the limitations imposed by the lack of a sugarcane genome sequence, only the cleavage of the diphenol oxidase laccase (TC122593) mRNA by miR408 could be confirmed by modified 5′ RACE. Curiously, eight out of 10 clones were cleaved near to the 5′ end of miRNA, in a position distant from the described canonical slicer sites (Figure 9d). 

## 3. Discussion

Recent studies have highlighted the regulatory role of non-coding RNA, like miRNAs, as a complex mechanism to respond to biotic stress [15,41,42]. In our study, biotic stress was induced in sugarcane by infection with *A. avenae*, the causal agent of Red Stripe Disease. This disease is an important bacterial disease, affecting many crops around the word [43]. The symptoms of the infection are red stripes and top rot of leaves [44,45]. The bacterium enters sugarcane leaves through the stomata and invades the intercellular spaces, but it does not reach the xylem and phloem vessels [46]. Despite of the losses of productivity caused by the infection with *A. avenae* in sugarcane, little is known about the molecular mechanisms triggered in sugarcane by this disease [7]. Data generated from the characterization of sRNAs and the regulatory network of the host’s immune systems need to be explored in order to develop tools to enhance plant resistance against this pathogen [41].

As a first step, a global view of the sRNAome was obtained by the analysis of sRNA libraries. Bioinformatics analyses of sugarcane sRNA libraries identified 25 miRNAs families of conserved miRNAs. Moreover, using the miRCat pipeline, 131 novel miRNAs were also identified that have lengths of 21 and 22 nt in our sugarcane libraries, increasing the number of novel sugarcane miRNA identified [33]. miRCat is considered an accurate method for the identification of novel miRNA [47]. However, only new miRNAs that have more than 35 reads in each library were considered. In addition, we evaluated the precursors structure, including the MFEI value, which showed that a majority of novel miRNAs precursors have an MFEI of more than 0.7, superior to tRNA, rRNA, and mRNA [35]. Finally, based on criteria for the annotation of miRNA [48,49], the novel miRNA that showed miRNA and miRNA* sequences identified in the same library were considered as bona fide precursors. Novel miRNAs can be classified as non-conserved miRNAs, which originate from recently evolved microRNA genes (MIR genes) [50]. 

The other class of sRNA, siRNA, was also identified in our analysis. Using data from the transcriptome of sugarcane infected with *A. avenae,* we observed that 110 differentially expressed transcripts showed more than 30 aligned siRNAs, suggesting that the expression of these transcripts can be regulated by siRNAs. One of these transcripts is a Ctr copper transporter, which is a member of a group of plasma membrane proteins or lysosome membrane proteins that mediate copper (Cu) uptake. Although copper is an essential micronutrient for plants, an excess of Cu is considered a toxic element as it generates hydroxyl radicals [28]. In rice, *Xanthomonas oryzae* overcomes plant defenses by regulating host copper redistribution, promoting the removal of copper from xylem vessels, and increasing the intracellular concentration in the shoots [51]. It is also noteworthy that several pesticides carry copper as a component, and that plant pathogens are sensitive to increased copper levels. Analyses of the sugarcane transcriptome show an induction of Ctr copper transporter in the presence of *A. avenae*. On the other hand, analyses of siRNA expression showed that siRNAs aligned with the Ctr copper transporter were downregulated in the presence of a pathogen. Interestingly, the alignment of a cluster of siRNAs occurred preferentially in the 5′ UTR of this transcript, in agreement with a report that showed that clusters of siRNAs aligned near to the core promoter and upstream from regions within 200 nt of the 5′ start sites of protein-coding genes [52]. One hypothesis is that the repression of the cluster of siRNAs that aligned here can lead to the induction of copper transporter. In addition, we observed that mock-inoculated plants accumulate more 24-nt siRNAs than 21/22-nt siRNAs. In plants, gene silencing can occur by DNA methylation guided by 24-nt siRNAs [53], suggesting that in non-inoculated plants, the copper transporter is regulated at the transcriptional level by RNA-directed DNA methylation. In contrast, in sugarcane infected with *A. avenae*, the upregulation of Ctr copper transporter occurs due to the repression of the siRNA regulating this transcript. This regulation can result in an increase of intracellular Cu content, similar to what was observed in rice overexpressing the copper transporter [51], while a study of the function of COPT1, a type of copper transporter, in *Arabidopsis* showed that COPT1 antisense plants had a decrease of Cu uptake and consequently lower Cu levels [54]. Curiously, some miRNAs, called Cu-miRNAs (miR397, miR398, miR408, and miR857), are also involved in copper homeostasis, targeting genes that encode Cu proteins [28]. The expression of these Cu-miRNAs can be regulated in response to Cu availability. For instance, low Cu results in the upregulation of these miRNAs, while with elevated levels of Cu these miRNAs are downregulated [26,55].

Several of the canonical sugarcane miRNAs were identified as differentially expressed in mock and *A. avenae*-infected libraries. Surprisingly, a sizable number of miRNA* was also found. However, even though both mature and miRNA* strands are produced in equal amounts by the processing of MIR gene transcripts, their accumulation is asymmetric at the steady state [56]. Although the miRNA families identified in replicates of sRNAs libraries are the same, only 10 miRNAs shared the same expression profile. The variability in the measurements observed between biological replicates can be influenced by the technical noise and the biological variation [57]. However, it is important to mention that the infection of the plants was confirmed by phenotypic analysis, bacterial colonization, and profile expression of pathogenesis-related genes in both biological replicates. In addition, the Pearson correlation test between mock and infected replicates showed cor > 0.82, suggesting a good correlation. Among the miRNAs that showed the same regulation profile in both replicas, miR408, a Cu-miRNA, is the most expressed miRNA, and it is downregulated in both replicates. The repression of miR408 was confirmed by qRT-PCR in the samples used for sequencing and in four additional biological replicas from an independent experiment, indicating that miR408 is downregulated upon pathogenic infection. Similar results were previously observed in Arabidopsis plants infected with pathogenic bacteria [58]. Moreover, as mentioned above, this result is in agreement with the increased expression of the copper transporter in sugarcane infected with *A. avenae*, eventually leading to an increase of Cu concentration. Finally, this would lead to the downregulation of Cu-miRNAs, including miR408.

In addition, 67 lincRNA candidates were identified using mRNAseq data from sugarcane infected with *A. avenae*. Similarly, lncRNA was described as being responsive to *Fusarium oxysporum* infection in *Arabidopsis thaliana*, highlighting the importance of 20 lincRNAs in plant defense networks [24]. In maize, 34 lincRNAs were recently predicted as miRNA targets and 86 lincRNAs appeared to function as miRNA decoys [59]. An example of a miRNA regulated network that also involves lncRNAs is the *IPS1* (Induced by Phosphate Satarvation 1), a non-protein-coding gene, complementary to miR399, with a 3-nt central mismatch. This mismatch suggests that miR399 does not provoke the cleavage of *IPS1*, but that the lncRNA acts to scavenge the miRNA, consequently decreasing the regulation of the canonical target, PHOSPHATE 2 (PHO2), a protein involved in the maintenance of phosphate homeostasis [60]. Here, we showed that miR408 aligns with an lincRNA, suggesting that this lincRNA could be acting as an miR408 target or decoy, adding a new layer of regulation. 

Plants can also establish beneficial associations with a wide range of microorganisms that colonize root surfaces or intercellular spaces [61]. Studies have shown that sugarcane establishes efficient interactions with beneficial diazotrophic bacteria [62,63]. When beneficial endophytic diazotrophic bacteria are recognized by sugarcane, they trigger a response in the plants that is unlike those directed to pathogenic bacteria. For instance, the SHR5 gene, a receptor-like kinase, is downregulated in sugarcane plants associated with beneficial endophytic bacteria but not upon infection with different pathogens [64]. In addition, the expression pattern of a putative ethylene receptor (SCER1) and two putative ERF transcription factors (SCERF1 and SCERF2) showed exclusive modulation in plants inoculated with the diazotrophic endophytes [65]. It was proposed that microorganisms have the potential to modulate the steady-state level of a number of miRNAs [66,67,68], which could be an indication of a significant role of miRNAs in the response of plants to microbial invasion. Depending on the type of plant-microbe interaction, certain miRNAs could be differentially modulated [69,70]. Here, we show that sugarcane miR408 was downregulated in plants infected with two different pathogens—*A. avenae* and *P. kuehnii*—suggesting that these miRNAs are generally involved in the response to biotic stress, regardless of the pathogen involved. Accordingly, a similar regulation of miR408 was observed not only for interaction with other bacteria and fungi, but also for interaction with viruses. In contrast, miR408 was upregulated in hybrid and wild species of sugarcane inoculated with the beneficial bacteria *G. diazotrophicus*. In addition, miR408 was also upregulated in maize inoculated with another beneficial endophytic diazotrophic bacteria, *H. seropedicae* [71], strengthening the evidence that the regulation of miR408 expression is correlated with the type of plant-microbe interaction, either beneficial or pathogenic. 

Recent reports have clearly demonstrated that plant miRNA can modify the expression of genes involved in plant-microbe interactions [71,72,73]. miR408 directs the cleavage and downregulation of an mRNA encoding a laccase [26]. PCR analysis demonstrated that the expression levels of two putative targets of sugarcane miR408 were inversely correlated with the expression of this miRNA, suggesting that the mRNAs encoding diphenol oxidase laccase and blue copper are likely targets of miR408. Interestingly, the targets of miR408 were found to be downregulated in sugarcane inoculated with a beneficial diazotrophic bacterium, while miR408 was upregulated in response to the presence of this bacterium. Although the repression of laccase was not observed for all sugarcane plants used, the cleavage of the diphenol oxidase laccase mRNA by miR408 was also confirmed using 5′ RACE. Laccase is a multicopper enzyme involved in diverse roles in plants, including lignin synthesis, browning, and wound healing [74,75].

**Figure 10:**
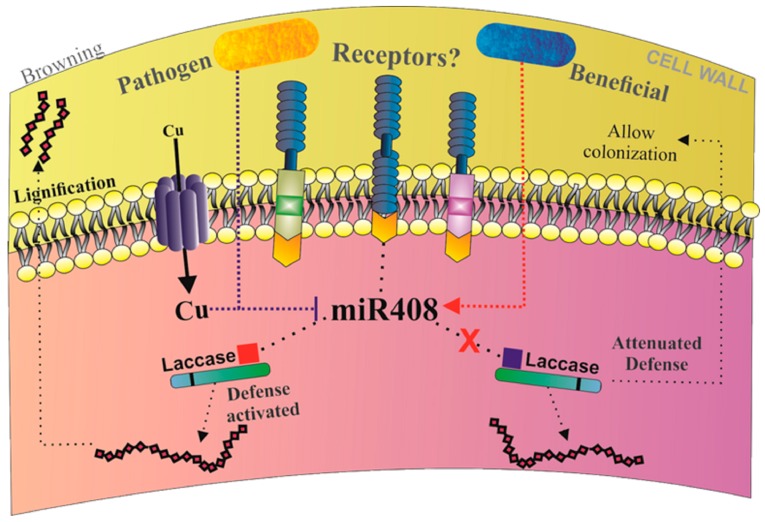
Model of sugarcane miR408 regulation in response to pathogen and beneficial endophytic diazotrophic bacteria. Pathogens attack sugarcane and trigger an upregulation of copper transporter as well as an increase in copper inside the cell resulting in a repression of miR408; consequently, the level of its targets increases. This regulation can result in cell wall lignification and browning. In contrast, the upregulation of miR408 can result in beneficial diazotrophic bacteria colonization by the cleavage of laccase.

The differential expression of miR408 in response to either pathogenic or beneficial microorganisms suggests a possible model for miRNA-mediated post-transcriptional regulation in response to biotic interactions (Figure 10). Once receptors of sugarcane sense the pathogen, a molecular response is triggered to combat infection. An early response to biotic stress is the accumulation of reactive oxygen species—ROS [76]. ROS can be formed in the presence of redox active metals, like Cu^+^ [77]. miR408 is involved in the control of regulatory networks that allow adaptation to the changing availability of copper, and its expression is downregulated under conditions of high copper concentration [26,78]. Therefore, one possible model would be that, upon pathogenic infection, there is an increase in copper levels due the downregulation of a cluster of siRNA, thus leading to increase in the expression of copper transporter. Consequently, in this situation the repression of miR408, a Cu-miRNA, occurs, and the miR408 target genes are induced in order to promote physiological and metabolic adaptation. Based on the 5′ RACE analysis, the target cleaved by miR408 is a laccase, an enzyme that can be involved in lignification and browning, two plant processes that play important roles in host defense against pathogenic invasion [79,80]. The increase in diphenol oxidase laccase mRNA levels could be explained by the involvement of the enzyme in the polymerization of phenolic compounds, which would protect plants from pathogen attack. While levels of lignin have not been measured in the experiments described here, brownish spots were clearly visible in plants infected with *A. avenae*. In contrast, in sugarcane inoculations with a beneficial microorganism, phenotypic responses typical of pathogenic attack were not observed, indicating that the plant does not recognize diazotrophic endophyte bacteria as a threat [64,65]. Accordingly, it has been shown recently that improved Agrobacterium-mediated transformation in monocots is obtained when plant defenses are attenuated [81].

## 4. Materials and Methods

### 4.1. Pathogen Infection Assay

*Acidovorax avenae* subsp. *avenae* was obtained from the Culture Collection of the Instituto Biológico and grown in NA medium (beef extract 3 g/L; Peptone 5 g/L; NaCl 5 g/L) at 28 °C. Sugarcane plantlets grown in vitro were maintained at 28 °C with an irradiance of 60 µmol photons m^−2^ s^−1^ and a 12-h photoperiod. After the development of a root system, pathogen-free plants were propagated as individual plants. A set of plants was inoculated by rubbing a suspension of *A. avenae* in distilled water (10^6^ CFU mL^−1^) in leaf injury and immersing the root system for 5 min in this bacteria solution, after which they were washed with distilled water in order to eliminate superficial bacteria. Another set was used as a mock-infected group. Inoculated and control plants were transferred to Murashige and Skoog (MS) medium and kept there for 7 days. After, whole plants were harvested and examined macroscopically for disease spots. Two biological replicas (Rep 1 and Rep 2) were prepared from both mock and infected plants and sent for sequencing. In addition, four more biological replicates (Rep 3, Rep 4, Rep 5, and Rep 6) were investigated to validate the expression profile of some miRNAs.

Plants infected with the fungi *Puccinia kuehnii* were selected from field trials based on the severity of visual infection symptoms of the disease, ranging from low to severe symptoms. From the same trials, asymptomatic plants were harvested as controls. For each treatment, leaves from four plants were collected.

### 4.2. Validation of A. avenae Infection

In order to validate the experiment, three analyses were performed: phenotypic, bacterial colonization counts, and expression of PR genes. Bacterial colonization was validated by plate counting using the Most Probable Number estimation [82]. The expression of PR genes was performed using the following steps: Total RNA were extracted with Trizol (Invitrogen, Carlsbad, CA, USA) and treated with DNaseI (Promega, Fitchburg, WI, USA). Total RNA was then reverse transcribed into cDNA using Super-Script III reverse transcriptase (Invitrogen). To analyze the expression profile, qRT-PCR was used with SYBR Green PCR Master Mix (Thermo Fisher Scientific, Waltham, MA, USA) in Applied Biosystems 7500 Real-Time PCR Systems (Thermo Fisher Scientific). To each well, 1 µL of first strand cDNA, 5 µL of SYBR Green solution, 2 µL of the forward primer (10 µM), and 2 µL of the reverse primer (10 µM) were added. Ct (cycle threshold) calculations were performed using 7500 Software v.2.0.5, and the relative expression was calculated [83]. Glyceraldehyde 3-phosphate dehydrogenase (GAPDH) and 28S, which are sugarcane housekeeping genes [84,85,86], were used as the internal controls. Primers used are available in Table S7. 

### 4.3. Treatments with Beneficial Diazotrophic Bacteria

Four SP70-1143 rooted sugarcane plantlets grown in vitro were transferred to a hydroponic system containing 0.5× Hoagland’s solution [87], and left for acclimatization over a period of 7 days. All plants were maintained at 30 °C with an irradiance of 60 µmol photons m^−2^ s^−1^ and a photoperiod of 12 h. Plants were then inoculated with the beneficial diazotrophic bacterium *Gluconacetobacter diazotrophicus*—PAL 5, following methods described by Vargas et al. [88]. Fourteen days after the inoculation, plants mock-inoculated or pathogen-infected were harvested, and RNA was extracted. In the experiment with parental sugarcane species, *S. barberi* and *S. officinarum*, plants were maintained under the same conditions, but harvested 7 days after inoculation.

### 4.4. RNA Extraction and Sequencing Small RNA Library Construction

Total RNA was isolated from whole plants using Trizol (Invitrogen) as described by the manufacturer. Total RNA (~10 µg) from control and pathogen-infected plants was sent to Fasteris Life Sciences SA (Plan-les-Ouates, Geneva, Switzerland) for the construction of sRNA libraries and subsequent sequencing with Illumina technology. In brief, the 20–28 fractions of total RNA were size selected, and unmodified RNA was used for library construction using an in-house developed protocol. After sequencing, the sequences were evaluated by measuring the quality of the reads according to previous reports [71]. The sequencing data were deposited in the NCBI Gene Expression Omnibus [89] under accession number GSE42628.

### 4.5. Bioinformatics Analysis

#### 4.5.1. Identification of sRNAs

Small RNA reads were trimmed and filtered if they had an exact full-length match to known plant tRNA or rRNA sequences or low-complexity sequences. Using the UEA sRNA toolkit—plant version filter pipeline (http://srna-tools.cmp.uea.ac.uk/) [90] with three different databases (plant t/rRNAs from Rfam, Arabidopsis tRNAs from The Genomic tRNA Database, and plant t/rRNA sequences from EMBL (European Molecular Biology Laboratory), reads with low-complexity (less than three different bases) and both sense and antisense matches with t/rRNAs were removed. The remaining reads were then analyzed to identify novel and conserved miRNAs and siRNAs. The UEA sRNA toolkit—plant version was used to identify novel and conserved miRNAs. The miRProf pipeline was used to identify conserved miRNA. This approach matches sRNA libraries with known Viridiplantae mature and miRNA* deposited in miRBase database release 21 [91], using the PatMaN program. Novel miRNAs were identified by the miRCat pipeline using sequences mapped to the available sugarcane sequence [34] to find clusters of sRNA. Only new miRNA with 21 and 22 nt and that showed more than 25 reads were analyzed. In addition, putative siRNAs were aligned, allowing one mismatch, in transcripts differentially expressed from the sugarcane transcriptome using plants infected with *A. avenae* [30].

To allow comparison between libraries, counts of miRNA families and siRNAs were normalized in reads per 1 million (RPM) and the amount of filtered reads, after removing low-quality reads, were used for normalization. Fold change was calculated to show which sRNAs were up- or downregulated. miRNA target prediction was performed using the Plant Small RNA Target Analysis Server, psRNATarget [92] with the *Saccharum officinarum* DFCI Gene Index (Release 3) as the reference dataset.

#### 4.5.2. Identification of lincRNA

Using data from sugarcane transcriptome datasets [30], lincRNAs of sugarcane were identified with a pipeline available in Figure S1, including a specific designed SVM (Support Vector Machine) model [32]. The pipeline was constructed using data from *Sorghum bicolor*, a plant evolutionarily close to sugarcane for which the full genome sequence is known. As a first step, coding sequences were removed using BLAST and the databases Repbase and PlantGDB. BLAST and the CantataDB database were the used to find sugarcane transcripts classified as lncRNAs. The sugarcane transcripts classified as lncRNAs were mapped onto the sorghum genome (downloaded from the PlantGDB database) to find those mapping to intergenic positions. In this step, the sugarcane transcripts classified as protein-coding and as lncRNAs were mapped using the software Segemehl [93]. Those lncRNAs mapping to regions between two genes were considered good lincRNA candidates. An SVM model was also used to identify sugarcane lincRNAs, and the output from this model was compared with that obtained by direct sequence analysis. In order to generate this model, the training positive dataset was built with lncRNAs from the CantataDB database, since there were too few sugarcane lncRNAs to build the model. The negative dataset was built using sugarcane transcripts already classified as protein-coding, with the same number of sequences used in the positive dataset.

In order to estimate whether these lincRNAs could be miRNA targets or decoys, sequences of lincRNAs were aligned with miRNAs using the UEA_sRNA_Workbench alignment tool, allowing up to three mismatches and one gap.

### 4.6. Validation of miRNA and Target Gene Expression by qRT-PCR

The expression profiles of six sugarcane mature miRNAs were assayed by stem–loop reverse transcription-PCR using RNA from the replicas that were sequenced [94,95]. In order to confirm the initial validation, a new experiment with four biological replicas was prepared and the expression of two copper-microRNAs—miR397 and miR408—as well the abundant miRNA159 were verified. To analyze the expression profile of mature miRNA, qRT-PCR was also used with the SYBR Green PCR Master Mix (Applied Biosystems, Foster City, CA, USA) in the conditions described above. To analyze the expression of computational identified miR408 targets, qRT-PCR was used with cDNA derived from Rep 1 as a template. The primers used are available in Table S7.

### 4.7. Semi-Quantitative RT-PCR

The expression profiles of sugarcane plants inoculated with several microorganisms were analyzed by semi-quantitative RT-PCR. These samples were obtained in a previous experiment [64,65]. Samples from plants inoculated with beneficial and pathogenic bacteria were collected 7 days after inoculation. PCR primers were designed to amplify precursors of mature miR408, miR164, and miR172 (Table S7). The first-strand cDNA reaction diluted four times was used in standard PCR reactions (5 µL PCR buffer without MgCl_2_, 1.5 mM MgCl_2_, 200 µM dNTP, 200 µM forward primer, 200 µM reverse primer, 1 Unit of Taq Polymerase (Eppendorf, Hamburg, Germany). PCR conditions were 94 °C for 1 min, followed by 32 cycles (94 °C for 15 s, 58 °C for 30 s, 72 °C for 30 s), and with 72 °C for 5 min. The actin constitutive gene was used as an internal control in PCR reactions, being amplified for 26 cycles. Products of the PCR reactions were eletrophoretically separated on 1% agarose gel, visualized with ethidium bromide under UV light, and then photographed. 

### 4.8. Modified 5′ RACE Assay

To confirm the cleaved targets by miR408, 5′ RACE was performed using the GeneRacer kit (full-length, RNA ligase-mediated rapid amplification of 5′ and 3′ cDNA ends, RLM-RACE, Invitrogen^®^) for mapping the 5′ end of an miR408 target, TC122593, predicted to encode a diphenol oxidase laccase. Briefly, RNA (5 µg) from sugarcane plants was ligated to a 5′ RACE adaptor. Random hexamer primers were then used for cDNA synthesis. PCR amplification of a cDNA fragment containing the cleavage site of the targets was carried out by nested PCR. Primers used in PCR are available in Table S7. RACE fragments were cloned into a pGEM T-easy vector (Invitrogen^®^) and sequenced.

## 5. Conclusions

Our results suggest that sugarcane, and perhaps other grasses, has developed a mechanism to differentially recognize microorganisms that are either beneficial or detrimental to growth. The data presented here implicate sRNA expression as part of this mechanism. In particular, miR408 is downregulated in response to pathogen infection but upregulated in the presence of beneficial bacteria. Further dissection of the role of miR408-mediated regulation of copper binding proteins could help to establish a model of response to pathogenic attack by sugarcane and related monocot plants. 

## Figures and Tables

**Table 1:** Summary of sequencing data from the small RNA library.

Total	Replicate 1	
	Mock	Infected
All reads	2,209,310	2,852,027
t/rRNA filtering ^1^	1,714,978	1,668,744
Low quality reads filtering ^2^	1,560,245	1,436,225
Conserved miRNAs ^3^	105,279	82,728
Unique		
t/rRNA filtering ^1^	930,266	802,581
Low quality reads ^2^	912,871	780,141
Conserved miRNAs ^3^	1872	2313
Novel miRNAs ^4^	86	67
Putative siRNA ^5^	910,913	777,761

^1^ Filtering for tRNA, rRNA, trimming for “N” bases and 3′ adapters; ^2^ Filtering for low-complexity sequence; ^3^ Known miRNAs deposited in the miRBase database; ^4^ Novel miRNA of 21 and 22 nucleotides (nt) in length and with more than 25 reads identified by the miRCat pipeline; ^5^ The remainder of sRNA that were not classified as novel or conserved miRNA.

## Supplementary Materials

The following are available online at www.mdpi.com/2311-553X/3/4/25/s1, Figure S1: Pipeline for identification of sugarcane lincRNAs, Figure S2: Heatmap of miRNA expression levels from bioinformatics analysis, Figure S3: Regulation of miR408 in response to different microorganisms, Figure S4: Regulation of blue copper by miR408, Table S1: Summary of sequencing data from Rep 2 of the small RNA library, Table S2: Results of the pipeline to identify lincRNAs in sugarcane, Table S3: List of novel sugarcane miRNAs with lengths of 21 and 22 nt, Table S4: Putative targets of novel miRNAs in sugarcane, Table S5: siRNA that aligned in sugarcane transcriptome, Table S6: Differential expression levels of conserved miRNAs with more than 35 raw reads in at least one library, Table S7: Oligonucleotides employed as primers or probes and the corresponding sequences.
